# ‘Reservoir dogs’: The emerging zoonotic risk associated with European dog imports to the UK

**DOI:** 10.1002/vetr.70551

**Published:** 2026-04-02

**Authors:** Poppy Simonson, Tapan Bhattacharyya, Michael A. Miles

**Affiliations:** ^1^ Department of Infection Biology London School of Hygiene and Tropical Medicine London UK

**Keywords:** dog travel, one health, vector‐borne disease, zoonoses

## Abstract

**Background:**

The movement of dogs from continental Europe to the UK poses a growing public health threat due to the associated risk of disease incursions. Current legislation is insufficient to address the risks and pre‐import control measures are focused only on rabies virus and the fox tapeworm *Echinococcus multilocularis*.

**Methods:**

We conducted a scoping review to summarise the major zoonotic pathogens and vectors associated with imported dogs (including rabies virus, *Brucella canis* and exotic tick species), and explore their potential to become established in the UK. Gaps in existing research and surveillance are highlighted, and potential measures to strengthen control are discussed.

**Limitations:**

Only English‐language sources were included in the literature search. Data on imported dogs and their disease burden are limited.

**Conclusions:**

Raising awareness of the risks among veterinary staff—who play a central role in recognising, managing and preventing imported zoonoses—is integral to a One Health approach.

## INTRODUCTION

The domestic dog (*Canis lupus familiaris*) co‐evolved alongside people and continues to provide social, health and economic benefits in the UK and worldwide.^1^ However, this close relationship carries public health risks. Dogs serve as hosts and reservoirs for many zoonotic pathogens, and they bring these pathogens and their vectors into human proximity, facilitating disease spread.

Several zoonotic pathogens and vectors have a lower or absent UK burden compared mainland Europe.^2^ Proximity and relative freedom of movement facilitate high dog import rates from Europe to the UK each year.^3^, ^4^ Current legislation specifies pre‐import control measures for only rabies virus and the fox tapeworm *Echinococcus multilocularis*, and there is no requirement for tick treatment. This enables introduction of non‐endemic pathogens and vectors of veterinary and public health concern via dog travel. Frequent subclinical canine infection and lack of screening mean that outbreaks may be detected at a later stage when intervention is more challenging. Veterinary staff and dog owners are often underinformed of this risk.^5^

While previous studies highlight the dangers of imported canine diseases to UK dogs,^2^, ^6^, ^7^, ^8^, ^9^ fewer focus on the zoonotic threat.^10^ Here, we explore the public health risk presented by infectious diseases and vectors carried by dogs travelling from continental Europe to the UK, identify current research gaps and discuss potential improved control measures.

## METHODS

We conducted a literature review of zoonotic pathogens and vectors relevant to European dog imports to the UK. Literature searches were conducted on PubMed, with search terms, including combinations of keywords relating to dog travel, location and zoonotic pathogens or vectors. Species of interest were explored with further searches, and reference lists of relevant publications appraised for secondary sources. We included English‐language publications between 2000 and 2025, comprising peer‐reviewed research and reviews, surveillance reports and some grey literature from governments and organisations. In addition, a freedom of information request was submitted to Defra (FOI2025/19899) regarding dog import numbers from 2018 to 2024.

## RESULTS

### Current status of UK dog imports

**FIGURE 1:**
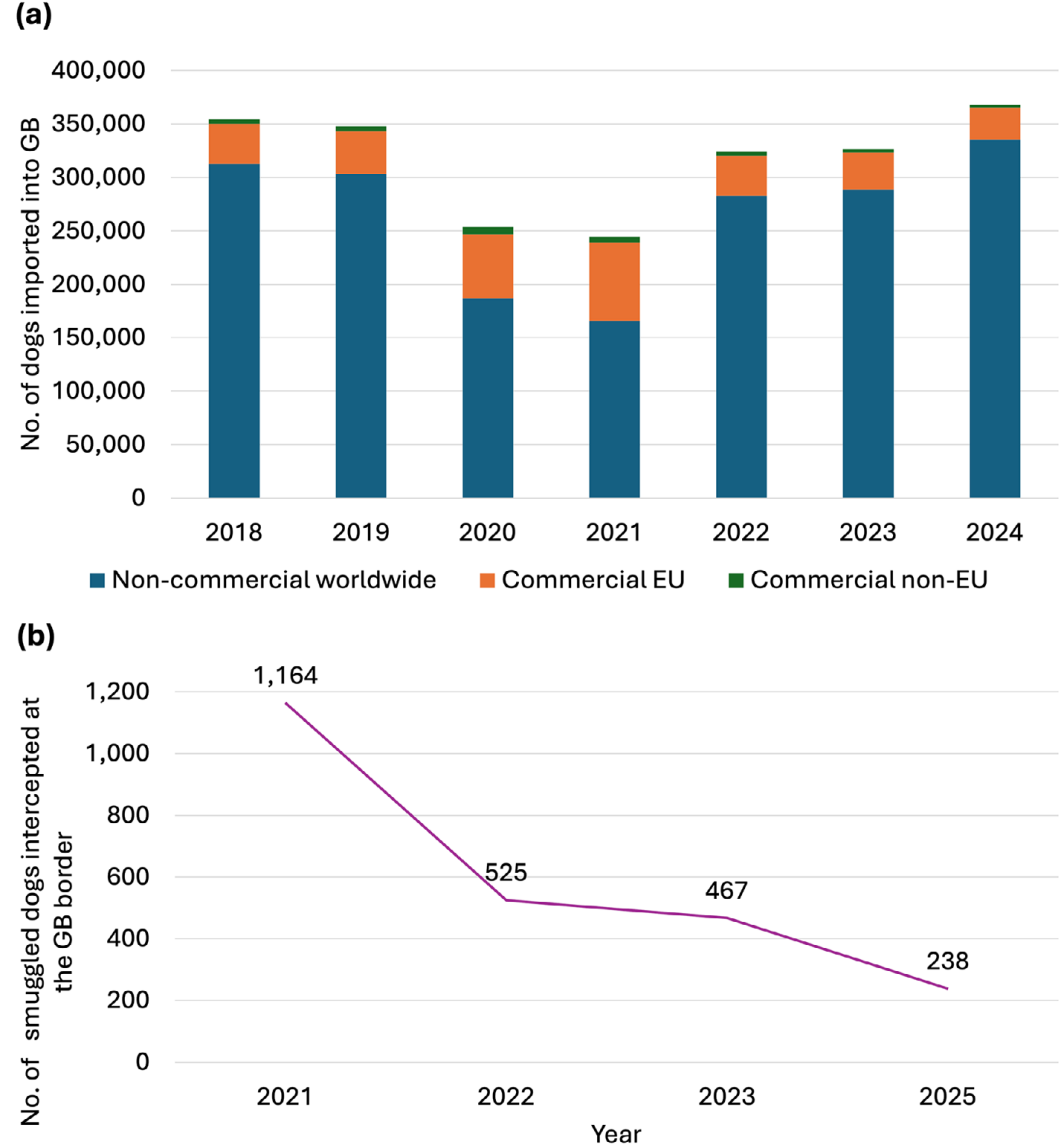
(a) Dog imports to Great Britain (GB) in 2018‒2025.^3^, ^4^ Due to collection methods, the data are related to GB rather than the UK, and non‐commercial imports are not subdivided by origin. Commercial imports comprise pedigree dog sales and charity imports; non‐commercial imports are typically pets travelling with owners, although some charities may incorrectly import rescues non‐commercially.^11^ (b) Numbers of illegally imported dogs intercepted at the GB border in 2021‒2025.^4^

There are currently around 8.5 million dogs in the UK,^11^ with an annual demand for almost one million new puppies.^5^ Following a reduction during the COVID‐19 pandemic, dog import rates have remained high (Figure 1a). In 2024, there were 335,451 non‐commercial (pet) imports to Britain, almost twice as many as in 2021 and more than pre‐pandemic numbers.

Dogs imported from Europe can be categorised as follows:

- Resident UK dogs returning from holiday with their owners:

The relative ease of taking a pet dog to nearby Europe is attractive to many. Unfortunately, owners are frequently underinformed about the importance of disease and vector prevention, and a small proportion of these dogs will return with a non‐endemic disease. Some pathogens, such as the protozoan *Leishmania infantum*, cause more severe disease in UK dogs, which lack immunity compared with endemic canine populations.^12^

- Stray dogs for adoption, imported by charities and other organisations:

Many organisations facilitate the adoption of street dogs, mainly from Eastern Europe. UK dog imports from Romania increased fivefold between 2014 and 2019.^13^ This facilitates spread of pathogens such as *Brucella canis*, among other health and welfare concerns.^14^ There is wide variation in pre‐export veterinary care and disease screening and in owner awareness of risk.^5^ Some may arrive with poor health status. For example, one cohort had high owner‐reported pathogen and ectoparasite burdens.^11^

- Commercial pedigree imports, imported by individual breeders and large businesses:

Increasing rates of obtaining pedigree puppies and dogs from abroad are likely due to the relative ease of breeding overseas, with rising UK dog prices and high demand.^5^ These dogs often cross multiple borders from areas of higher infectious disease prevalence.^5^ The importation of pregnant bitches and dogs from large breeding establishments raises particular concern for *B. canis* transmission.^14^

- Illegal imports, smuggled by individuals or organised crime groups:

Lax breeding regulations and high demand for pedigree puppies facilitate the illegal dog trade in Europe.^15^ The scale of dog smuggling is difficult to estimate, but a survey conducted in 2025 found that 20% of UK small animal veterinary surgeons had seen a suspected illegally imported puppy in the previous 12 months.^16^ The numbers of intercepted dogs have reduced significantly since 2021 (Figure 1b). However, without evidence to suggest a true reduction in smuggling, this likely represents a lack of interception.^4^ Smuggled dogs present a high disease risk because they are often unsuitably young, without rabies vaccination or tapeworm treatment, and may be from areas of higher disease burden.^15^

Factors determining the rate of UK dog imports and associated disease risk are outlined below:

- Legislation:

Currently, imported dogs are subject to control for rabies virus and *E. multilocularis*. All dogs entering the UK from Europe must be at least 15 weeks old, with a microchip and rabies vaccination. Deworming treatment is required unless entering from another *E. multilocularis*‐free country.^17^ In 2012, requirements for rabies vaccine serology testing (for low‐risk countries) and tick treatment were removed. Although we lack data on the effectiveness of these control measures, their removal may have increased infectious disease risk.^6^ Current regulations are likely insufficient to prevent the entry of rabies virus, *E. multilocularis* and many other pathogens that are subject to no controls. Lack of mandatory tick treatment may also allow introduction of vectors to dogs.^18^ Infectious disease risk is also affected by inadequate regulation of dog imports and measures to control the illegal dog trade.

- Sociopolitical factors:

Societal and political trends, such as increased demand during the COVID‐19 pandemic,^19^, ^20^ influence the number and type of dogs imported. Barriers to UK adoption may stimulate the sourcing of dogs from overseas. Reasons cited for adopting a rescue dog from abroad include responding to online marketing, perceived poor welfare, concern that the dog may be killed and failure to meet criteria of UK rescues.^11^ Geopolitical events, such as displacement of dogs with owners due to conflict, also affect risk.^21^

- Environmental factors:

Climate‐induced expansion of many vector species is predicted to continue. Temperature increases of 1°C‒2°C can have wide‐reaching effects, including altered transmission patterns of UK‐endemic vectors and emergence of new species.^22^ Further expansion of the distribution and active season of endemic *Ixodes ricinus* (tick vector of *Borrelia burgdorferi* and other pathogens) is expected.^23^ Recent years have seen UK emergence of mosquito vectors of *Dirofilaria* species and the fly vector of *Thelazia callipaeda*, with predicted pathogen emergence.^24^, ^25^ Milder temperatures have allowed expansion of the phlebotomine sandfly (vector of *Leishmania* species) to northern Europe.^26^, ^27^ Establishment of invasive ticks, such as *Rhipicephalus sanguineus*, is also more likely as temperatures rise.^28^

### Imported canine pathogens presenting a zoonotic risk

The zoonotic pathogens most relevant to dog importation from continental Europe to the UK are summarised in Table 1 and the text below. We included pathogens absent from the UK or with a substantial prevalence differential compared to Europe, such that importation would meaningfully alter zoonotic risk. *Anaplasma phagocytophilium* and *B. burgdorferi*, although endemic in the UK, are included due to higher European prevalence.^29^ We excluded many pathogens (e.g., *Toxocara canis*, *Leptospira* species and *Giardia intestinalis*) with established high prevalence in resident dogs. *Babesia* species are not discussed as the European zoonotic species lack a canine reservoir.^30^

**TABLE 1:** Pathogens endemic in Europe that may be imported to the UK via dogs.

Pathogen	Vector	Epidemiological role (dogs)	European endemicity	UK disease status (dogs)
Viruses				
Rabies virus	None	Reservoir^31^	Parts of Eastern Europe^32^	Eliminated^32^
Tick‐borne encephalitis virus	*Ixodes* ticks; *I. ricinus* primary vector in Europe and endemic in the UK^33^	Accidental host^34^	Most of Europe^35^	Potential sporadic autochthonous^36^
Bacteria				
*Anaplasma phagocytophilium*	*Ixodes* ticks; *I. ricinus* primary vector in Europe and endemic in the UK^33^	Potential reservoir^37^	Most of Europe^37^, ^38^	Emerging^37^
*Borrelia burgdorferi*	*Ixodes* ticks; *I. ricinus* primary vector in Europe and endemic in UK^33^	Accidental host^23^	Most of Europe^23^	Emerging^23^
*Brucella canis*	None	Reservoir^39^	Most of Europe^14^	Sporadic imported^40^
*Ehrlichia canis*, *Ehrlichia chaffeensis*	*Rhipicephalus sanguineus* ticks; emerging in UK^33^	Reservoir (*E. canis*), potential reservoir (*E. chaffeensis*)^23^	*E. canis* in Mediterranean Europe^38^ *E. chaffeensis* emerging^23^	Sporadic imported and autochthonous^41^, ^42^
*Francisella tularensis*	Ticks (including *I. ricinus*, *Dermacentor reticulatus*, likely others) and mosquitoes, particularly *Aedes* species; several vectors endemic in UK^43^	Accidental host^43^	Most of Europe^44^	Absent^45^
*Rickettsia conorii*	*R. sanguineus* ticks^46^; emerging in UK^33^	Potential reservoir^23^	Mediterranean Europe^47^	Sporadic imported^48^
Protozoa				
*Leishmania infantum*	*Phlebotomus* species sandflies in Europe; absent in UK^49^	Reservoir^26^	Mediterranean Europe^26^	Sporadic imported and autochthonous^50^, ^51^, ^52^, ^53^
Helminths				
*Dirofilaria immitis*, *Dirofilaria repens*	Mosquito genera *Anopheles*, *Culex*, *Aedes* and others^54^; several species endemic in the UK^55^	Reservoir and definitive host^56^, ^57^	Mediterranean Europe^54^	Sporadic imported^57^
*Echinococcus multilocularis*	None	Reservoir and definitive host^10^	Most of Europe^27^	Absent^44^
*Thelazia callipaeda*	Fly *Phortica variegata*; present in southern UK^25^	Reservoir and definitive host^25^	Most of Europe^58^	Sporadic imported^58^
Arthropods				
*Linguatula serrata*	None	Reservoir and definitive host^59^	Parts of Europe^59^	Sporadic imported and autochthonous^60^

*Note*: Prevalence and distribution are likely underestimated due to limitations in data and diagnostic tests. Many dogs harbour coinfection due to factors such as common vectors and poor health status.^29^, ^61^ Definitions: reservoir—species in which a pathogen is maintained and transmitted to a defined group (here, people); accidental host—species which may be infected without meaningfully contributing to onward transmission; definitive host—species in which adult parasites mature and reproduce sexually (vs. intermediate host, in which immature stages develop).^62^, ^63^

Dogs can influence zoonotic transmission through several epidemiological pathways. They may directly introduce pathogens for which they are reservoirs, such as *B. canis*,^40^ or accidental hosts (see Table 1 footnote for definitions). Dogs also function as transport hosts for vectors, including ticks, increasing opportunities for human exposure to vector‐borne pathogens.^30^ For several pathogens, such as *B. burgdorferi*, canine cases are sentinels for human disease.^64^

### Viruses

#### Rabies

Rabies, caused by *Lyssavirus* species, results in progressive neurological signs and is almost uniformly deadly once signs develop.^31^ Classical (non‐bat) rabies is most commonly transmitted to people by dog bites, facilitated by the virus's induction of aggression in many infected dogs.^32^

Vaccination of animal reservoirs (dogs and wild carnivores) has significantly reduced the incidence of classical rabies in Europe.^65^ Most countries have achieved elimination, including the UK, which has been rabies‐free since 1922.^66^ Western Europe has seen sporadic imported cases,^32^ with subsequent localised outbreaks in Italy and Greece.^67^ All imported animal cases since 2000 were due to illegal movements.^66^ The virus persists in parts of Eastern Europe due to spillover from bordering endemic countries,^32^, ^65^, ^68^ with a fatal autochthonous human case in Romania in 2025.^69^

Importation of an animal with rabies is the most likely mode of UK introduction.^66^ Pets from low‐risk countries must wait 3 weeks after rabies vaccination before entering the UK; for high‐risk countries, a rabies vaccine serology test and a 3‐month wait are also required.^70^ All European countries are categorised as low risk, although not all are rabies free.

Concerns arise from this approach. Vaccine failures have been reported.^71^, ^72^ Additionally, the long viral incubation period may allow importation of incubating cases. In 2008, a dog imported from Sri Lanka was diagnosed with rabies in a UK quarantine facility.^73^, ^74^ Unvaccinated imported dogs are quarantined for an inadequate 3 weeks post‐vaccination.^6^ Illegally imported dogs, which are commonly unvaccinated and enter unidentified, present the highest risk. A naïve reservoir population, including dogs (not routinely vaccinated unless travelling) and wildlife, means that UK endemicity is possible without swift detection of imported cases.^32^

#### Tick‐borne encephalitis

Neurotropic tick‐borne encephalitis virus (TBEV) is spread predominantly by *I. ricinus* in Europe and has diverse wildlife reservoirs.^34^ Dogs are a rare accidental host and may display progressive neurological signs, including paralysis and seizures.^36^ While most human TBEV infections are asymptomatic, severe neurological signs may result.^75^

TBEV infection is endemic in Europe (estimated incidence of 2.19 cases per 100,000), with an expanding distribution.^35^ The virus has recently been detected in ticks and people in the UK,^75^ with potential canine cases, although similarity to endemic Louping Ill virus complicates diagnosis in dogs.^36^, ^75^ The tick vector (*I. ricinus*) and many sylvatic reservoirs are endemic in the UK,^76^ although public health risk and potential for wider spread are considered low.^77^

### Bacteria

#### Anaplasma phagocytophilium

*A. phagocytophilium* causes human and canine granulocytic anaplasmosis. Spread by *Ixodes* ticks, its animal hosts include dogs.^78^ Infection may result in fever, joint pain and low white blood cell and platelet counts in people and dogs.^23^ However, many infected dogs are asymptomatic.^79^

*A. phagocytophilium* is endemic across most of Europe and emerging in the UK.^38^ The primary vector, *I. ricinus*, is the predominant UK tick species.^37^ The prevalence of *A. phagocytophilium* was 4.6% among ticks collected from one cohort of UK dogs.^37^ The UK also has many other potential reservoirs, including wild and domestic ruminants.^78^

#### Borrelia burgdorferi

These spirochaete bacteria have diverse wildlife reservoirs, and their primary European vector is *I. ricinus*.^33^, ^80^ In people, following the classic ‘bullseye’ rash, symptomatic Lyme disease presents as fever, joint pain and neurological signs.^23^ Dogs are accidental hosts and may develop similar signs to people or have asymptomatic carriage.^81^

Lyme disease is endemic across most of mainland Europe, with an annual incidence of around 128,000 cases and much local variation.^82^, ^83^, ^84^ It is emerging in the UK, with regional hotspots in Scotland and southern England.^85^ UK prevalence is likely underestimated and expected to increase as climate change extends the vector's active season.^64^, ^86^ The mean prevalence of *B. burgdorferi* in British ticks is estimated to be 3.6% (vs. 12.3% in Europe).^23^, ^87^ Two studies found prevalences of 0.5% and 2.0%, respectively, in ticks removed from UK pet dogs.^64^, ^88^

#### Brucella canis

*B. canis* is one of several species causing brucellosis in people and animals. It is transmitted principally via reproductive secretions from dogs, its primary reservoir.^89^ Infected dogs may display chronic and wide‐ranging signs, including discospondylitis, infertility and abortion.^14^ In people, severe cases develop meningitis and septicaemia.^90^ Asymptomatic carrier status is common in both people and dogs.^14^, ^90^ Innate antibiotic resistance necessitates prolonged courses in people.^91^ Infection is almost impossible to cure in dogs; hence, euthanasia is often advised.^92^

*B. canis* is endemic in dogs in Eastern Europe.^14^ Rare human cases occur, mainly in those with compromised immune systems or prolonged close contact with dogs.^92^ Human and canine prevalence are likely underestimated due to lack of screening.^89^

**FIGURE 2:**
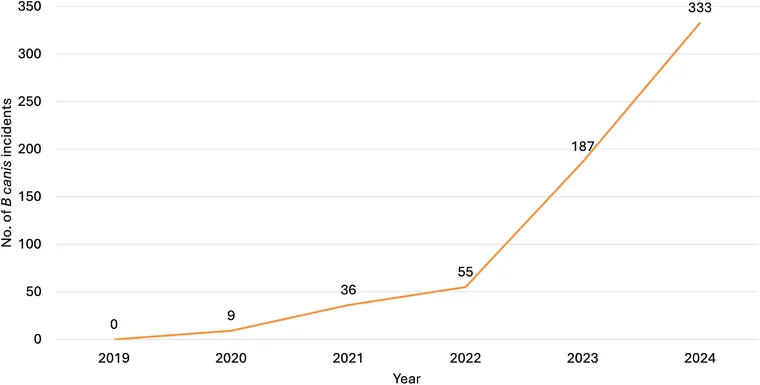
Incidence of *Brucella canis* in UK dogs in 2019‒2024.^92^, ^94^, ^95^ Lack of routine surveillance means these numbers are likely underestimated.

In the UK, *B. canis* is emerging in dogs. Before 2020, just three imported canine cases had been confirmed.^92^ However, incidence has risen sharply since 2020 (Figure 2), likely due to rising dog imports from endemic countries such as Romania and no mandatory pre‐import screening.^90^, ^92^ Autochthonous dog‐to‐dog spread is now reported.^90^ The first zoonotic transmission occurred in 2022, from a pregnant rescue dog imported from Belarus to its immunocompromised fosterer,^93^ with one additional case since in a veterinary staff member.^90^

UK zoonotic risk is considered low but is higher for dog owners, immunocompromised people and veterinary staff.^90^ Thus far, person‐to‐person spread has not been reported.^90^, ^92^ *B. canis* has been reportable since 2022, while other *Brucella* species are notifiable and under stricter controls due to higher zoonotic risk.^92^ In late 2025, Defra announced dogs that are commercially imported from Romania must be screened for *B. canis* before entering the UK.^96^

#### Ehrlichia canis and Ehrlichia chaffeensis

Spread by *R. sanguineus*, these bacterial species cause human and canine monocytic ehrlichiosis. In dogs, signs include fever, lethargy and coagulation disorders; rare human infection has similar symptoms.^46^

While *E. canis* is widely distributed in dogs across Europe, human cases are uncommon.^97^ *E. chaffeensis* is emerging in people and dogs in Europe.^23^ However, serological cross‐reactivity complicates the interpretation of species prevalence.^23^ The UK sees sporadic imported canine *E. canis* cases, and in 2012, two autochthonous cases were reported in untravelled dogs in southeast England.^41^ *E. chaffeensis* has not been reported in the UK to date.^23^ Recent UK detection of *R. sanguineus* may facilitate pathogen emergence, although likelihood of establishment is low.^98^

#### Francisella tularensis

In people, tularaemia causes fever, muscle pain and lymphadenopathy, with potential severe complications.^43^ Transmission routes include vectorial (mosquitoes and ticks), direct contact with animal reservoirs and environmental exposure.^33^, ^43^, ^46^ In animals, severity varies from asymptomatic carriage to peracute illness and death.^43^ Dogs are considered an accidental host but may transmit to people given close proximity, and occupational exposure is a risk factor.^43^, ^99^, ^100^

Tularaemia is endemic across most of Europe, with an average incidence of 0‒17.9 cases per 100,000 reported between 1997 and 2013.^43^ While tularaemia is considered absent from the UK,^45^ a 2023 human case without recent travel history raised concerns about autochthonous exposure from an animal or tick.^45^ Epidemic or endemic spread is possible if *F. tularensis* was imported, given its contagiousness and presence of environmental and wildlife reservoirs.^45^, ^101^ However, the probability of establishment is unknown.

#### Rickettsia conorii

*R. conorii*, spread by *R. sanguineus* ticks, causes Mediterranean spotted fever in dogs and people.^46^ Symptoms in people include ‘tache noire’ rash, fever and malaise, with potentially fatal complications.^23^ Underdiagnosis in dogs is likely due to frequent subclinical presentation.^46^ Contact with the canine reservoir is a risk factor for human infection.^23^

*R. conorii* is endemic across Mediterranean Europe, with a mean seroprevalence of 3.9%‒23% in people and up to 80% among dogs in one endemic area.^46^, ^47^ Recent emergence in people in Switzerland, Poland and Denmark has been linked with import of infected ticks on dogs.^44^ The UK has reported no autochthonous cases; however, the recent detection of the tick vector may allow future emergence.^98^

### Protozoa

#### Leishmania infantum

This parasite causes human and canine visceral leishmaniosis (CVL). Dogs are the principal reservoir.^102^ It is predominantly spread by sandflies (*Phlebotomus* species)^103^; salivary, vertical and venereal transmission between dogs has also been reported.^104^, ^105^ In people, symptomatic infection (including fever, anaemia and splenomegaly) is nearly always fatal if untreated.^102^ In dogs, severity varies (from asymptomatic to severe skin disease, fever, weight loss, onychogryphosis and anaemia) and infection is typically lifelong.^106^ Treatment aims to manage clinical signs, reduce parasite load and improve quality of life, but it is rarely curative.

*L. infantum* and its sandfly vector are endemic in Mediterranean Europe, where the average CVL seroprevalence is 23.3%.^26^ The parasite is also emerging in northern Europe, due to dog movement and expanding vector distribution as a result of climate change.^26^, ^107^

Absence of the sandfly vector has prevented endemic CVL transmission in the UK.^50^ However, increasing imported cases have been reported, particularly in southeast England, although lack of surveillance hinders prevalence estimates.^50^ Three autochthonous CVL cases were diagnosed between 2018 and 2019 in untravelled dogs.^51^, ^52^, ^53^ Currently, potential for UK zoonotic transmission or significant autochthonous spread of *L. infantum* is extremely low due to vector absence. However, if warmer temperatures enable sandfly expansion to the UK, there is potential for endemicity due to the naïve canine reservoir population.^50^

### Helminths

#### Dirofilaria immitis and Dirofilaria repens

These filarial nematodes are spread by various mosquito species.^56^ Dogs are their primary reservoir, in which asymptomatic infection is common.^24^ Colonisation of the canine heart and pulmonary vasculature by *D. immitis* (also known as heartworm) may cause coughing and heart failure,^57^ while *D. repens* colonises the skin and may cause subcutaneous nodules.^56^ Human infections are rare and most commonly due to *D. repens*, with variable presentation.^56^

Both species are endemic in Mediterranean Europe and have expanded northwards in the past two decades.^57^ Pooled European canine *D. immitis* prevalence is around 10.5%.^54^ Neither species is UK endemic, but increasing imported canine cases are seen.^55^ There is potential for endemicity, particularly for *D. repens*, which has spread more rapidly through northern Europe.^24^ Several mosquito vectors are already present in the UK; the key vector *Aedes albopictus* has been detected sporadically, with endemicity predicted as temperatures rise.^55^, ^108^

#### Echinococcus multilocularis

In the intermediate human host, *E. multilocularis* causes hepatic alveolar echinococcosis, which is generally fatal without treatment.^44^ Asymptomatic animal reservoirs include dogs, foxes (definitive hosts) and wildlife.^27^

The parasite's range is expanding in Europe, except in a few non‐endemic countries, including the UK.^27^ European incidence of alveolar echinococcosis varies by country, averaging 0.21 cases per 100,000 in 2023.^27^

Dog travel is key for *E. multilocularis* spread, and is the most likely mode of UK introduction.^109^ Modelling suggests human cases are highly likely if pre‐entry deworming treatment was abandoned.^109^ The presence of several native sylvatic reservoirs means the parasite would be challenging to eradicate if it becomes established.^28^, ^44^ Prevention, via maintaining current controls and reducing illegal imports (likely without deworming treatment), is therefore vital.

#### Thelazia callipaeda

The eye worm nematode is spread by its fly vector and intermediate host, *Phortica variegata*.^58^ Adult nematodes colonise the canine and human conjunctiva, resulting in ocular signs.^25^ *T. callipaeda* has emerged across most of Europe in the past two decades, spreading as far north as France.^58^ The UK has seen sporadic imported canine cases since 2016.^58^ There is potential for endemicity, with vector presence in southern England (and potentially suitable conditions across much of UK), and potential sylvatic reservoirs.^25^

### Arthropods

#### Linguatula serrata

*L. serrata*, known as tongue worm, is a pentastomid parasite of mammals.^59^ Dogs are infected by consuming infective larvae in raw offal, and colonisation of the nasopharynx produces respiratory signs or asymptomatic carriage.^110^ Rare human infection occurs via raw meat ingestion or exposure to nasal discharge and faeces of infected dogs.^59^

*L. serrata* is infrequently reported in Europe, and prevalence data are limited.^59^ The UK has recently seen several imported cases in dogs from Romania^60^ and a single autochthonous case in a raw‐fed dog.^111^ Lack of surveillance may mean the zoonotic risk is underestimated.

### Imported vectors associated with dog imports

**FIGURE 3:**
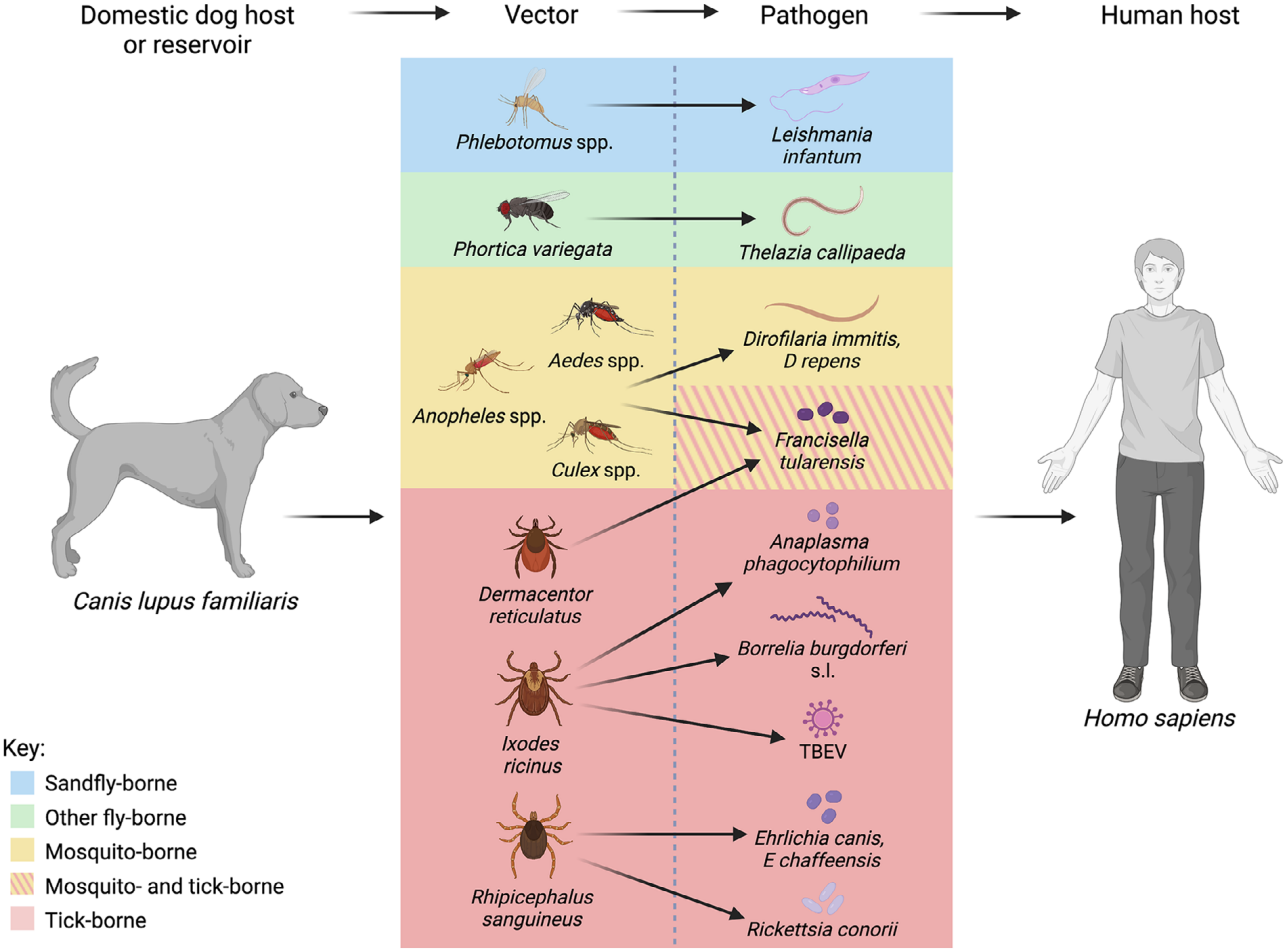
Schematic of vector‐borne zoonoses of dogs that pose a UK public health threat, created in BioRender. *Borrelia burgdorferi* and *Anaplasma phagocytophilium* are the two species currently established in the UK.^37^, ^85^

The vector‐borne canine zoonoses that are current or potential threats to the UK are summarised in Figure 3 and the text below.

Ticks are the main vectors of concern associated with dogs because of their direct attachment. Dogs play a key role in bringing infected ticks into proximity with people and wildlife reservoirs. Various tick species have emerged throughout continental Europe and the UK due to dog travel, climate change and other factors.^88^, ^112^, ^113^, ^114^ The abolishment of mandatory pre‐entry tick treatment may facilitate introduction of exotic species to the UK.^6^, ^44^ *I. ricinus* (vector of *A. phagocytophilium*, *B. burgdorferi*, *F. tularensis* and TBEV) is the predominant UK tick species.^115^ *R. sanguineus* (vector of *E. canis*, *E. chaffeensi* and *R. conorii*) has emerged since its introduction via dog travel in 2012.^98^ *R. sanguineus* and other non‐native ticks, such as *Dermacentor reticulatus* and *Hyalomma* species, have been identified on untravelled dogs in the UK, although establishment is unlikely at current temperatures.^2^, ^115^

Although not imported directly on dogs, fly vectors spread several zoonotic pathogens. The northwards expansion of sandflies (*Phlebotomus* species) is modelled to continue with climate change,^107^ with *L. infantum* emergence. *P. variegata*, the vector of *T. callipaeda*, is endemic in the southern UK and is predicted to expand.^25^ Finally, mosquitoes, including invasive *A. albopictus*, are associated with spread of pathogens, including *Dirofilaria* species.^2^

## CONCLUDING REMARKS AND FUTURE ACTIONS

There is a lack of disaggregated data on the numbers of imported dogs and their disease burden. The pathogens discussed herein have variable UK prevalence, and dogs play different roles in their spread. Currently, most are introduced sporadically without sustained onward transmission. Climate change has influenced the expansion of several European vectors and pathogens, including mosquitoes (for *Dirofilaria* species), sandflies (for *L. infantum*) and *R. sanguineus* (for *Ehrlichia* species). As climate change may exacerbate over half of human infectious diseases,^116^ further shifts in epidemiology are expected, facilitated by changing land use and urbanisation.^115^

With high dog numbers entering the UK from Europe, there is a growing need to consider the zoonotic implications. Imported dogs are not a homogenous group; most holidaying pet dogs present low risk, while illegally imported dogs and street dogs from Eastern Europe likely carry higher zoonotic risk.^42^, ^117^ The recent increase in imported *B. canis* cases with subsequent autochthonous transmission provides an important paradigm.

**FIGURE 4:**
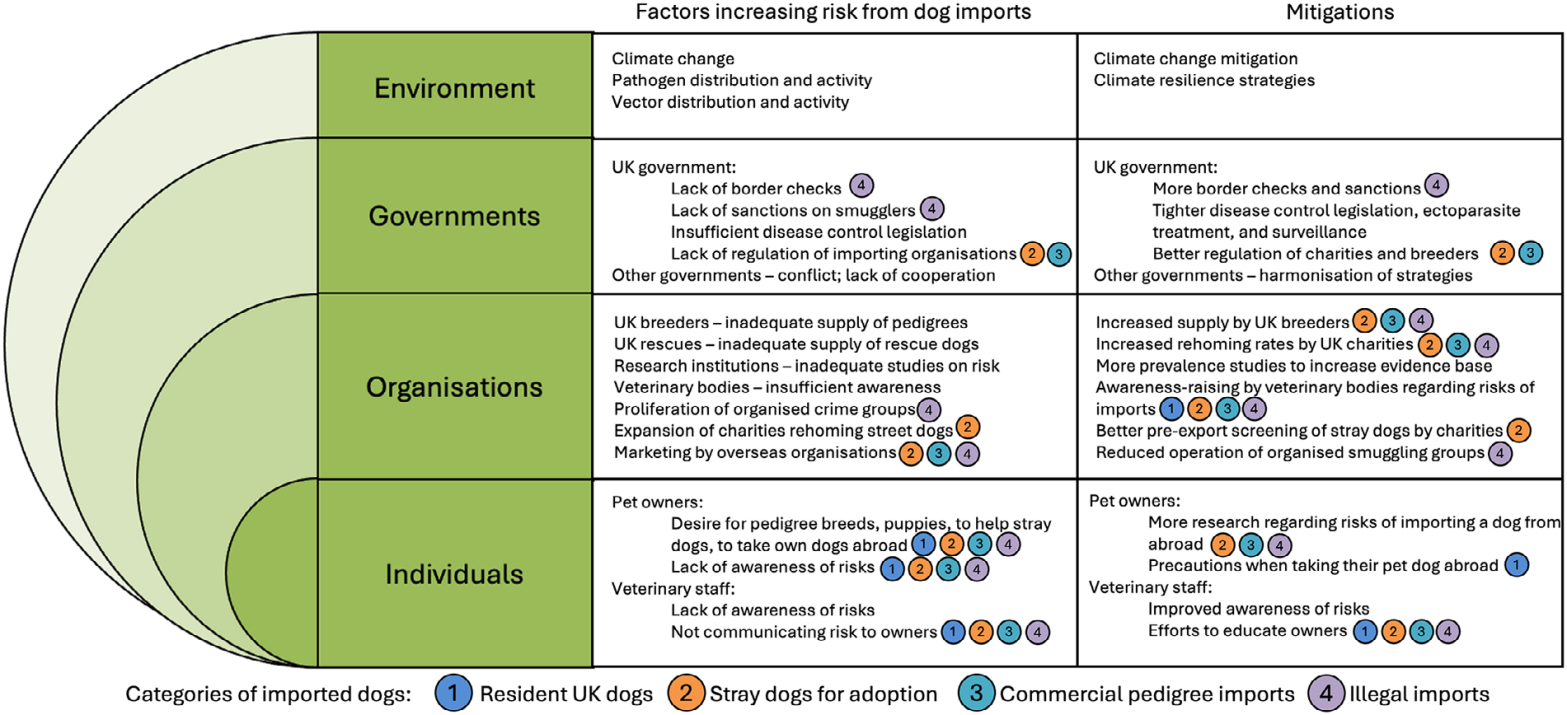
Schematic summarising current challenges to control of zoonoses introduced by dog travel to the UK and suggested mitigations.

As risk varies by pathogen and import pathway, targeted controls are likely more effective than blanket measures (Figure 4). Further research is required to inform evidence‐based policy, including cross‐sectional studies quantifying disease burden in resident imported dogs and those scheduled for importation. Given resource limitations, canine disease surveillance should be proportionate to zoonotic risk, and human surveillance tailored to high‐risk groups. Current initiatives, such as the tick surveillance scheme, are invaluable to clarify UK vector endemicity.^118^

Existing legislation should be reviewed and, where appropriate, updated in line with evidence. The UK's exit from the EU offers an opportunity to reassess biosecurity measures as an island nation with defined entry points. The BVA advocates for a UK‒EU agreement facilitating trade while allowing necessary divergence on biosecurity.^119^ Australia and New Zealand provide examples of non‐land bordered countries with more rigorous pre‐entry requirements.^120^, ^121^

Potential legal interventions could include the following:

- extension of the pre‐import waiting period after rabies vaccination from 3 weeks to 3 months;
- reintroduction of pre‐import tick treatment, informed by pilot studies to quantify public health benefit;
- pre‐import *B. canis* screening in all endemic areas;
- enhanced border checks to intercept illegally imported dogs;
- improved accountability for organisations importing high dog numbers, including and clearer health and welfare responsibility.

The impact of existing and future measures is contingent on their enforcement and compliance. Reforms in the Animal Welfare Act 2025, including increasing the minimum age of imported puppies, are a positive step.^122^ However, disease control and welfare benefits may be limited without parallel investment in border enforcement. The introduction of *B. canis* screening for commercial dog imports from Romania omits key groups, including misclassified rescue imports,^11^ pets travelling with owners, illegal imports and dogs from other endemic regions. Proposed interventions must also balance benefits against potential harms, such as the contribution of tick treatment to insecticide resistance and environmental contamination.^123^

Early detection and proportionate response remain central to control of emerging pathogens and vectors. Improved surveillance and further research are required to better quantify current and future zoonotic risks associated with canine imports from Europe. Vigilance among UK veterinary staff and a One Health approach (integrating veterinary, medical, research and policy sectors) are essential to ensure that these risks identified early and managed effectively.

## AUTHOR CONTRIBUTIONS

Poppy Simonson conceived the study, performed the literature searches and wrote the manuscript. Tapan Bhattacharyya and Michael A. Miles advised on the study scope and direction and revised drafts of the manuscript.

## CONFLICT OF INTEREST STATEMENT

The authors declare they have no conflicts of interest.

## FUNDING INFORMATION

The authors received no specific funding for this work.

## ETHICS STATEMENT

Ethical approval was not required as this was a review of the existing literature and no new data were generated.

## Data Availability

Data sharing is not applicable to this article as no datasets were generated or analysed during the current study.
